# Assessing childhood maltreatment exposure using the child behavior checklist

**DOI:** 10.3389/frcha.2025.1493432

**Published:** 2025-05-08

**Authors:** Takuya Makino, Shota Nishitani, Shinichiro Takiguchi, Akiko Yao, Takashi X. Fujisawa, Akemi Tomoda

**Affiliations:** ^1^ Division of Developmental Higher Brain Functions, United Graduate School of Child Development, The University of Osaka, Kanazawa University, Hamamatsu University School of Medicine, Chiba University, University of Fukui, Osaka, Japan; ^2^ Research Center for Child Mental Development, University of Fukui, Fukui, Japan; ^3^ Life Science Innovation Center, University of Fukui, Fukui, Japan; ^4^ Department of Child and Adolescent Psychological Medicine, University of Fukui Hospital, Fukui, Japan

**Keywords:** child abuse, child behavior checklist, childhood maltreatment, ROC curve analysis, sensitive analyses

## Abstract

**Introduction:**

Childhood maltreatment (CM) has broad and severe adverse effects in later life, but there are not enough studies conducted during childhood close to the time of maltreatment. Most studies have focused only on a single symptom and have not attempted to capture the global picture of CM.

**Methods:**

We used the Child Behavior Checklist (CBCL) to assess children's behavioral/emotional problems more comprehensively. This study leveraged 32 CM children and 29 typically developing (TD) children who have been assessed using the CBCL 4–18 from our dataset. Group comparisons of the eight subscales were conducted to characterize each behavioral/emotional problem. Receiver Operating Characteristic (ROC) curve analysis was conducted to assess the classification performance. Finally, sensitive period and type analyses were performed based on the children's maltreatment history.

**Results:**

The CM group showed significantly higher behavioral/emotional problems in seven out of the eight subscales. Logistic regression analysis was performed using all combinations of CBCL subscale T-scores and age, sex, and IQ. We created 2047 models and performed ROC analysis for each. Three models were generated: the most accurate model (comprising CBCL T-score, age, sex, and IQ; sensitivity: 0.906, specificity: 0.966), a model excluding IQ (sensitivity: 0.875, specificity: 0.931), and a model consisting only of CBCL (sensitivity: 0.906, specificity: 0.862). The CBCL demonstrated robust predictive capacity for CM by utilizing information provided by caregivers, without directly inquiring about trauma. The sensitive period analysis revealed that the temporal predictor of severity for “withdrawn” and “thought problems” were exposure to CM at age five. Similarly, exposure to CM between the ages of five and seven predicted “somatic complaints”. In the case of type, physical abuse was the predictor for “somatic complaints” and “delinquent behavior”, and emotional abuse was the predictor for “anxious/depressed” and “thought problems”.

**Conclusion:**

Maltreated children present a wider range of behavioral/emotional problems, which must be considered when supporting them. Perspectives gained from sensitive analyses of maltreatment history will help clinicians provide more appropriate interventions.

## 1 Introduction

Childhood maltreatment (CM) survivors have been shown to present with extensive and severe psychosocial problems in childhood as well as in adulthood (1). CM increases the risk of developing depression in adulthood that is less likely to remit (2, 3). It is also associated with a wide range of psychopathologies, including an increased prevalence of eating disorders (4) and personality disorders (5). The adverse impact of CM on later life is related not only to mental health but also to lifestyle-related diseases, such as obesity (6) and hypertension in sexually abused women (7). According to a large epidemiological study conducted by the Centers for Disease Control and Prevention (CDC) in the United States, a higher number of adverse childhood experiences leads to increased premature mortality risk in later life (8). In the context of social life, CM survivors also have fewer years of education, employment, assets, and income than those without CM exposure, highlighting how CM can negatively impact their quality of life later (9).

The majority of studies are retrospective studies of adults, and with exceptions such as the Bucharest Early Intervention Project (10), which examines the long-term brain effects of receiving institutional care vs. foster care, there are not enough studies on CM effects on childhood. The few existing studies involving children have reported that maltreated children showed physical and mental vulnerabilities, including asthma in physically and sexually abused children (11), deterioration of sleep (12), reduced social interaction, such as emotional recognition from the eyes (13). However, these are separate studies that have focused on each feature observed in maltreated children. In addition to clarifying the vulnerability of maltreated children to individual symptoms through individual hypotheses, research that takes a comprehensive approach to the overall picture of their vulnerability is also necessary.

Psychological scales that measure traumatic symptoms in children, including a short form of the Childhood Trauma Questionnaire (14) and Trauma Symptom Checklist for Children (15), have been used in clinical circumstances. However, the administration of these scales involves directly asking children about their maltreatment and traumatic experiences, which places a high psychological burden on them. The Child Behavior Checklist 4–18 (CBCL) is a non–self-rating questionnaire that parents and surrogate parents, in “problem items”, use to comprehensively rate behavioral/emotional problems in their children (16, 17). It has been used to characterize children with social anxiety (18), and the subscales have also been used in clinical trials of medications for children with conduct disorder (19).

Therefore, this study aimed to characterize the behavioral/emotional problems of maltreated children using the CBCL. The influence of maltreatment history, such as type of maltreatment and length, on behavioral/emotional problems was also compared. Among the various traits that were comprehensively captured, those that were particularly relevant to CM were extracted and their feasibility as predictors for CM was also examined.

## 2 Materials and methods

### 2.1 Participants

Thirty-two children with maltreatment experiences (CM group) aged 8–16, and 29 typically developing children with no history of maltreatment (TD group) aged 10–17 participated in the study. Many children from the CM group were separated from their biological parents by the Child Protection Service or its equivalent and placed in residential childcare facilities. At the time of this study, the children participating in the study and receiving social care were in residential institutional care. Most of them were in small groups, with two children in large-scale group. All children were in a protected environment (20). All children in the CM group experienced either physical, emotional, or sexual abuse or neglect (ICD-10-CM Code T74). The Child Protection Service's records are based on objective observations and assessments, with the provision of a safe environment. This is important for reliability and accuracy in maltreatment diagnoses and interventions. Individual records were continuously collected and updated over time by various professionals (e.g., behavioral evaluations by temporary shelter staff, medical evaluations by child mental health specialists and pediatric nurses, social evaluations by Child Protection Service staff, and psychological evaluations by licensed psychologists). Detailed demographic characteristics are shown in Table 1. Participants' intelligence quotient (IQ) was measured with the Wechsler Intelligence Scale for Children-Fourth Edition (WISC-IV) or Wechsler Adult Intelligence Scale-Third Edition (WAIS-III). One participant in the CM group had a specific learning disorder with no IQ problem (IQ = 81). Our study included this participant because it was confirmed that they had experienced maltreatment. The clinical diagnostics for neurodevelopmental disorders were assessed by a pediatric psychiatry clinician.

**Table 1:** Sociodemographic characteristics.

	CM (*n* = 32)	TD (*n* = 29)	Statistics	*p* value
Male participants, *n* (%)	24 (75.0)	20 (69.0)	*Χ*^2^ (1) = 0.28	0.60
Age (years), Mean (SD)	11.7 (2.1)	13.1 (2.1)	*t* = −2.59	0.01
Type of maltreatment, *n* (%)				
Physical abuse	20 (62.5)	–		
Emotional abuse	23 (71.9)	-		
Neglect	27 (84.4)	–		
Sexual abuse	4 (12.5)	–		
Duration (years) of maltreatment, Mean (SD)*	7.9 (4.1)	–		
FSIQ, Mean (SD)	91.3 (12.4)	108.4 (9.7)	*t* = 6.01	1.33E-07
WISC-Ⅳ	91.3 (12.4)	108.1 (9.40)	*t* = 5.90	2.19E-07
WAIS-Ⅲ	–	112.5 (17.68)	–	–

* Including during pregnancy.

CM, childhood maltreatment; TD, typically developing; SD, standard deviation; FSIQ, full scale intelligence quotient.

The protocol of this study was approved by The Research Ethics Committee of University of Fukui (Assurance no. 20220039) and was conducted in accordance with the Declaration of Helsinki. This study made secondary use of data obtained from previous studies (21, 22). The study was conducted using an opt-out method in lieu of obtaining written consent, and opt-out information was published on the website of the University of Fukui Hospital in Japan.

### 2.2 Child behavior checklist (CBCL) 4–18

The parent-rated version of the CBCL has been used to measure children's behavioral/emotional problems (16). In the case of CM, the staff in charge of the child welfare facility, foster parents, or their parents not involved in maltreatment conducted the ratings. The checklist has subscales, including “withdrawn”, “somatic complaints”, “anxious/depressed”, “social problems”, “thought problems”, “attention problems”, “delinquent behavior”, and “aggressive behavior” (16). These eight subscale scores represent the severity of the problem in each domain. Standardized T-scores were used in each domain. In contrast to the original version, the Japanese version of the CBCL was designed to make it applicable for 4–15-year-old children (23). The T-score for the five participants (CM: 1, TD: 4) older than 15 years was calculated as if they were 15 years old since there was no conversion table for that age.

### 2.3 Receiver operating characteristic (ROC) curve analysis for classification model

Both stepwise regression and Lasso regression were examined to create a model for predicting CM. Stepwise regression uses the statistical information criterion for selecting a regression model. However, this model may not consistently exhibit a high area under the curve (AUC) or Youden index in receiver operating characteristic (ROC) analysis. Similarly, Lasso regression has the same consistency problem. Additionally, Lasso regression is concerned about reproducibility because it uses random numbers in its calculations. Therefore, we chose to perform logistic regression analysis for all combinations of CBCL subscale T-scores and age, sex, and IQ. Using the predicted probabilities from each regression analysis, ROC analysis was performed to calculate the AUC and Youden index; models with high AUC and high Youden index were considered to determine the most suitable model for adoption. For practical reasons, models without IQ and with CBCL only were also created; the model with the highest Youden index was selected. If there were multiple models, the one with the highest AUC was chosen.

### 2.4 Sensitive period and type analyses for maltreatment history

As in our previous study (24), the sensitive period in which CM exposure may be more strongly associated with behavioral/emotional problems was explored using random forest regression with conditional inference trees (“cforest” in R package party) (25). Two separate analyses were conducted to assess the importance of potential predictors for behavioral/emotional problems. In the first analysis, we evaluated the importance of specific periods of exposure, such as maternal domestic violence during pregnancy and exposure to neglect or physical, emotional, or sexual abuse from birth to age 18. Exposure to each type of maltreatment was coded as 0 (no exposure) or 1 (exposure). The second analysis assessed the importance of exposure to different forms of abuse (physical, emotional, sexual) and neglect, as well as the cumulative number of maltreatment types experienced. This cumulative maltreatment variable was treated as a continuous variable, representing the total number of maltreatment types each participant experienced. Each random forest model consisted of 200 trees, with 4 variables randomly selected at each node. These parameters were guided by theoretical considerations (26, 27) and follow common practices in similar prior research (24) and not optimized through model tuning by grid search. We used permutation testing to assess model performance, comparing the results from the original and permuted data to evaluate statistical significance. Variable importance was assessed using mean decrease in accuracy, where each variable's importance is based on the reduction in prediction accuracy when its values are permuted. This approach highlights the most influential variables in the model.

### 2.5 Statistical analysis

To compare the behavioral/emotional problems of the CM and TD groups, a multiple linear regression analysis was used for each of the eight domains. The explanatory variables were group (CM group was set to 1 and TD group was set to 0), age, sex, and full-scale IQ. The maximum likelihood with robust standard errors was employed as the estimator since the Breusch-Pagan test failed to assume homoscedasticity for some of the subscales. The Benjamini–Hochberg method was used to correct for false discovery rate (FDR) for multiple testing. All statistical analyses were performed using R software (version 4.4.1) (28) and packages eeptools (29), lmtest (30), lavaan (31), Epi (32), leaps (33), pROC (34), ggplot2 (35), and party (25, 36–39).

## 3 Results

### 3.1 Between-group comparisons

As shown in Table 2, the CM group showed significantly higher behavioral/emotional problems compared to the TD group in seven of the eight subscales (0.004 < *FDR* < 0.009), except for somatic complaints (*FDR* = 0.456).

**Table 2:** Effects of CM or TD group on each behavioral/emotional problem using multiple regression analysis.

	mean (SD)	range	β	SE	*z*-value	*p*-value	FDR
Withdrawn	58.81 (7.69)	50–75	0.41	2.12	2.664	0.008**	0.009**
	52.41 (3.97)	50–63					
Somatic complaints	52.78 (4.86)	50–68	0.10	1.15	0.745	0.456	0.456
	51.45 (3.5)	50–64					
Anxious/depressed	60.09 (9.06)	50–80	0.54	2.65	3.351	<0.001****	0.004***
	52.41 (4.76)	50–72					
Social problems	58.94 (6.82)	50–77	0.32	1.56	2.693	0.007**	0.009**
	52.24 (3.84)	50–65					
Thought problems	55 (9.14)	50–83	0.56	2.88	2.734	0.006**	0.009**
	50.83 (2.11)	50–56					
Attention problems	60.12 (8.28)	50–78	0.45	2.35	2.934	0.003***	0.008**
	51.34 (2.73)	50–61					
Delinquent behavior	59.66 (7.05)	50–70	0.42	2.06	2.861	0.004***	0.008**
	53.34 (7.05)	50–69					
Aggressive behavior	60.78 (8.73)	50–82	0.41	2.04	3.277	0.001***	0.004***
	52.66 (4.92)	50–71					

** *P* < 0.01.

*** *P* < 0.005.

**** *P* < 0.001.

The mean and range values for the CM group are in the upper row and those for the TD group are in the lower row.

Β, standardized regression coefficient; SE, standard error of the estimate.

### 3.2 ROC analysis for classification model

**Figure 1:**
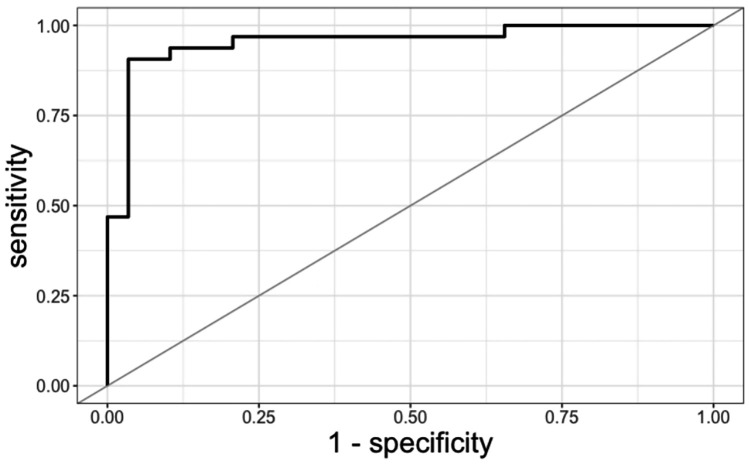
ROC curve of CBCL.

Model 1 in Table 3 displays the top model out of 2,047 total combinations, having high AUC and Youden index. Since the model with the highest AUC also had the highest Youden Index, it was chosen as the most accurate. This model had an AUC of 0.955 with a 95% CI of [0.901–1], a Youden index of 0.872, a sensitivity of 0.906, a specificity of 0.966, and a cutoff point of 0.599. The ROC curve is shown in Figure 1. Models without IQ, and only with CBCL, are also shown in Table 3. The coefficients for the three models are listed in Table 4. The T-scores for each CBCL subscale are multiplied by the coefficients, summed, and logit transformed. The resulting score was evaluated using the cutoff points outlined in Table 3.

**Table 3:** Models for predicting CM using the CBCL.

	Cutoff	AUC	Sensitivity	Specificity	Youden index
Model 1 Including the CBCL, age, sex, and IQ					
Somatic complaints, anxious/depressed, social problems, thought problems, attention problems, delinquent behavior, aggressive behavior, age, IQ	0.599	0.955	0.906	0.966	0.872
Model 2 Including the CBCL, age, and sex					
Withdrawn, anxious/depressed, thought problems, attention problems, age, sex	0.509	0.912	0.875	0.931	0.806
Model 3 Including only the CBCL					
Withdrawn, anxious/depressed, attention problems, delinquent behavior	0.384	0.898	0.906	0.862	0.768

**Table 4:** Coefficients for each model.

	Intercept	Withdrawn	Somatic complaints	Anxious/depressed	Social problems	Thought problems	Attention problems	Delinquent behavior	Aggressive behavior	Age	Sex	IQ
Model 1	0.292	—	−0.138	0.012	−0.073	0.231	0.217	0.057	0.043	−0.189	—	−0.167
Model 2	−15.045	0.111	−0.186	0.047	0.079	0.131	0.181	—	—	−0.390	—	—
Model 3	−22.098	0.127	−0.145	0.030	0.017	0.137	0.204	—	0.036	—	−0.305	—

### 3.3 Sensitive period and type analyses for maltreatment history

**Figure 2:**
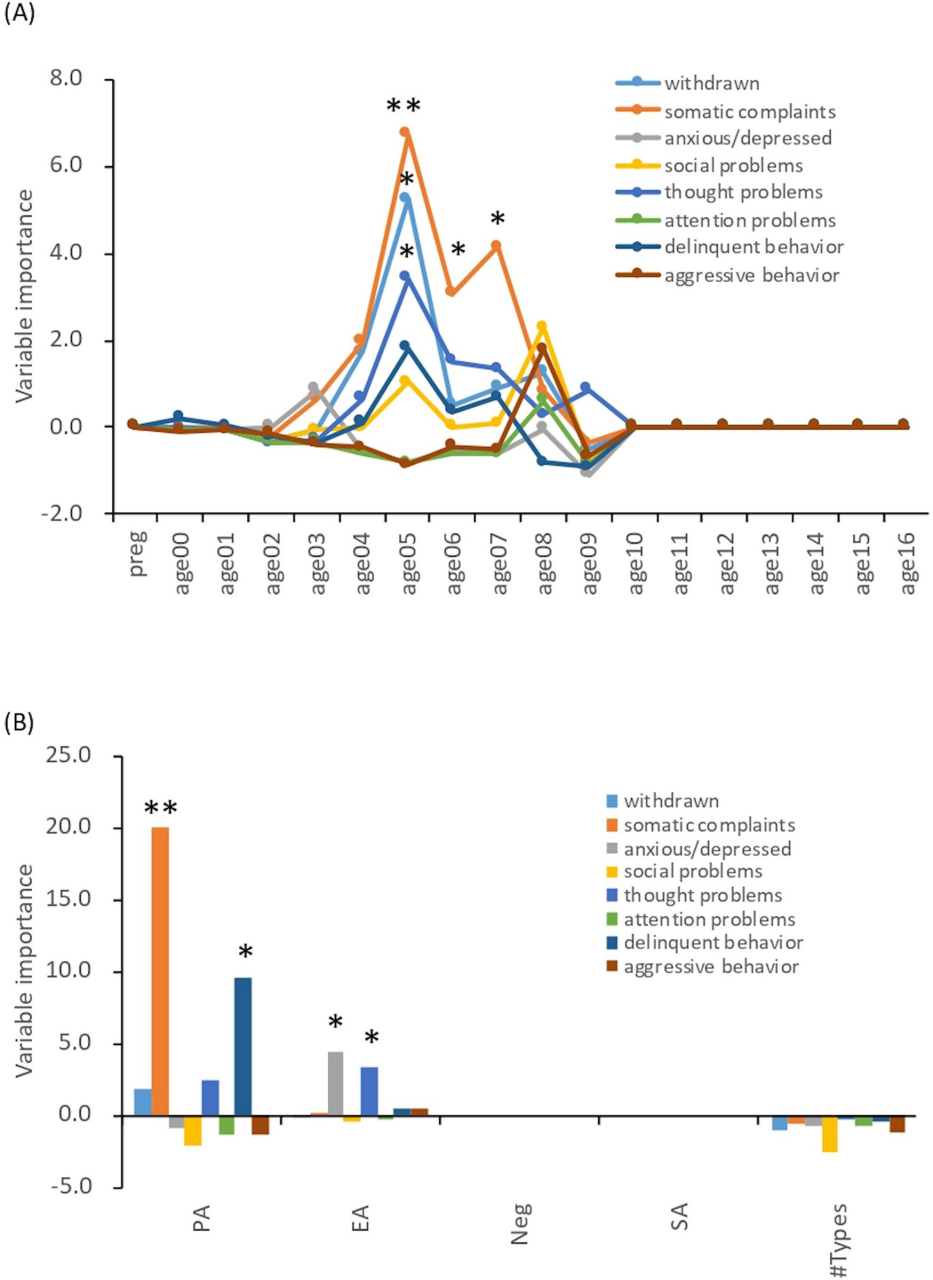
Sensitive period and type analyses. The data are based on historical records from the Child Protection Service or its equivalent agency up to the time of the CBCL evaluation. Of the CM group participants, 17 had experienced maltreatment since during pregnancy. Exposure to maltreatment was coded as 0 (no exposure) or 1 (exposure). **(A)** Sensitive period analyses. Preg: during pregnancy. *: *P* < 0.05, **: *P* < 0.01. **(B)** Sensitive type analyses. PA, physical abuse; EA, emotional abuse; Neg, neglect; SA, sexual abuse. *: *P* < 0.05, **: *P* < 0.01.

The most important temporal predictor of the severity of the somatic complaints domain was CM exposure at ages 5–7 years (peak at 5 years old) (Figure 2A, *MSE* = 82.56, *r* = 0.41, *Ps* < 0.05). Similarly, the withdrawn and thought problems domains were predicted by CM exposure at 5 years old (Figure 2A, *MSE* = 86.55, *r* = 0.35, *P* = 0.02 and *MSE* = 87.56, *r* = 0.34, *P* = 0.04). Symptom severity could be predicted with reasonable accuracy from the type of CM. One specific maltreatment, physical abuse (PA), was the most demanding predictor of somatic complaints and delinquent behavior (Figure 2B, *MSE* = 83.40, *r* = 0.38, *P* = 0.007, and *MSE* = 86.84, *r* = 0.33, *P* = 0.046, respectively). Emotional abuse (EA) emerged as the most important predictor of the anxious/depressed and thought problems domains (Figure 2B, *MSE* = 88.72, *r* = 0.32, *P* = 0.03 and *MSE* = 88.72, *r* = 0.32, *P* = 0.04, respectively).

## 4 Discussion

The aim of this study was to comprehensively evaluate the mental and physical features of maltreated children using the CBCL. The CM group scored higher than the TD group on seven subscales except for that of somatic complaints. Comparing the standardized regression coefficients, the subscales of thought problems, anxious/depressed, and attention problems were higher, in that order. CBCL thought problems consist of items screening obsessive-compulsive disorder (40) and/or psychotic symptoms (41), the raw scores on thought problems in CM revealed higher scores for items indicating obsessive thoughts and compulsive behavior. A previous study examined adult patients with obsessive-compulsive disorder who had CM experiences (42), and our results indicated that obsessive thoughts and compulsive behaviors are preceded during childhood. Our previous study using the DSRS-C, which measures depressive symptoms in children, showed that maltreated children presented significantly higher depressive symptom scores than the children in the TD group (21). The higher anxious/depressed scores in this study may be an analog for the higher depressive symptoms seen in the CM group. It has been repeatedly verified that CM causes sleep disturbances (43). Sleep disturbances, such as insomnia, are a major risk factor for daytime inattention (44); however, the CBCL does not have a subscale for sleep disturbances, but seven sleep-related items were used in previous study (45). The results of a group comparison by multiple linear regression analysis of the overall scores on these seven items showed that the CM group has a trend toward significant higher scores (*P* = 0.017). Thus, the inattention problems that were prominent in the CM children may have resulted partially from sleep disturbances. Regarding the assessment of sleep disturbance in children, CBCL did not cover it sufficiently and it would need to be measured more accurately with more specialized scales and objective methods such as actigraphs. For somatic complaints, there was no significant difference. Adolescents are prone to physical complaints such as headaches and abdominal pain even in normal circumstances (46), which may have made it difficult to detect differences. Therefore, the CBCL results appear to capture the characteristics of CM, yielding outcomes that do not dissociate from the practical clinical picture.

A model using the T-scores of the CBCL subscale, age, and IQ was found to predict CM with a high probability of AUC 0.955, sensitivity of 0.906, and specificity of 0.966 (47). This model may have the potential to capture features of maltreated children and be used for screening, even though it is not a questionnaire that directly asks about traumatic or maltreatment experiences. To the extent that CM features can even be captured from only a combination of comprehensive questions, in contrast to questionnaires that explicitly ask about traumatic or abusive experiences, CBCL may be useful and less psychologically invasive for screening for CM.

The different maltreatment histories in CM and their impact on the behavioral/emotional problems of maltreatment children were examined through a sensitive period analysis (24). The severity of being withdrawn and having thought problems were associated with maltreatment exposure at 5 years of age. This is a period of language development (48) and social development, when social skills gradually increase as children interact with others based on attachments (49). Maltreatment during this period may impede social development and the child may become withdrawn and develop thought problems in the future due to social isolation. Somatic complaints were significantly associated with whether the children had been exposed to maltreatment at ages 5–7, although somatic complaints comprised the only subscale that did not differ between the CM and TD groups. A possible explanation, although the exact reason is not known, is that the tendency for somatic complaints is due to immature language development (50, 51). In the CM group, the delay in language development due to CM may contribute to somatic complaints at the age of 5 to 7 years. Even in the TD group, nonverbal distress may have manifested as physical symptoms due to underdeveloped language. However, the factors influencing their language development remain unclear.

Analysis by type of abuse showed that PA was a predictor of somatic complaints and delinquent behavior. The importance of PA in the severity of somatic complaints may suggest that physical punishment, such as punching and kicking, can affect the development of brain regions involved in the control of somatic symptoms in children. Indeed, harsh corporal punishment during childhood reduced the prefrontal cortex gray matter volume in young adults, as reported by our group (52). Furthermore, increased delinquent behavior when subjected to active maltreatment, such as PA, was observed in previous studies (53). In addition, EA was important concerning the severity of being anxious/depressed and having thought problems. EA has been reported to increase anxiety and depressed symptoms (54). The EA group showed high scores for obsessive-compulsive symptoms (40), while scores for items measuring other psychotic symptoms were lower than that. According to previous studies (55), EA is associated with more severe obsessive-compulsive disorder symptoms in adult patients, and this finding may be consistent with previous ones.

There are several limitations to this study. First, because the CBCL is a questionnaire designed to be answered by parents and surrogate parents, the results may be inaccurate due to bias depending on the raters or information they did not have access to. Second, the study included two participants with IQs below 70. In addition, the CM group had significantly lower IQs than the TD group; it has been repeatedly shown (56) that children who have experienced CM have lower IQs than TD children, and the participants in this study may reflect these assumptions. Third, the Japanese version of the CBCL is standardized for ages 4 to 15. However, this study included participants aged 16 to 17, who were converted to T-scores as though they were 15 years old. This may affect the validity of the analysis. Fourth, the model to predict CM using T-scores of the CBCL subscale, age, and IQ showed very high predictive power, but has not been validated with other data. Thus, the model will need to be validated with larger sample sizes and cross-validation. Fifth, we did not perform any parameter optimization of the model, such as grid search, which could affect its performance and the accuracy of the results. We also used permutation testing to assess model performance and did not perform cross-validation; nevertheless, we acknowledge the potential value of such model optimization and performance assessments and plan to incorporate them in future work. Finally, a larger sample size might have made it possible to observe sex differences (e.g., somatic symptoms are likely to be more prevalent in girls).

## 5 Conclusion

Maltreated children exhibited various behavioral/emotional problems compared to TD children. Therefore, when providing supportive interventions, it is necessary to pay attention not only to their traumatic symptoms but also to existing behavioral/emotional problems. The CBCL is a useful questionnaire to comprehensively measure those underlying issues. Furthermore, we believe that the perspectives gained from a sensitive analysis of maltreatment history contribute to the establishment of more precise interventions.

## Data Availability

The datasets presented in this article are not readily available because ethical considerations. Requests to access the datasets should be directed to corresponding author. Data may be provided to interested researchers upon reasonable request to the corresponding authors, after clearance from The Research Ethics Committee of University of Fukui and consent of all authors.
